# Deep PPG: Large-Scale Heart Rate Estimation with Convolutional Neural Networks

**DOI:** 10.3390/s19143079

**Published:** 2019-07-12

**Authors:** Attila Reiss, Ina Indlekofer, Philip Schmidt, Kristof Van Laerhoven

**Affiliations:** 1 Robert Bosch GmbH, Robert-Bosch-Campus 1, 71272 Renningen, Germany; 2 Bosch Sensortec GmbH, Gerhard-Kindler-Straße 9, 72770 Reutlingen, Germany; 3 Ubiquitous Computing, University of Siegen, Hölderlinstr. 3, 57076 Siegen, Germany

**Keywords:** heart rate, PPG, dataset, time-frequency spectrum, deep learning, CNN, evaluation methods

## Abstract

Photoplethysmography (PPG)-based continuous heart rate monitoring is essential in a number of domains, e.g., for healthcare or fitness applications. Recently, methods based on time-frequency spectra emerged to address the challenges of motion artefact compensation. However, existing approaches are highly parametrised and optimised for specific scenarios of small, public datasets. We address this fragmentation by contributing research into the robustness and generalisation capabilities of PPG-based heart rate estimation approaches. First, we introduce a novel large-scale dataset (called *PPG-DaLiA*), including a wide range of activities performed under close to real-life conditions. Second, we extend a state-of-the-art algorithm, significantly improving its performance on several datasets. Third, we introduce deep learning to this domain, and investigate various convolutional neural network architectures. Our end-to-end learning approach takes the time-frequency spectra of synchronised PPG- and accelerometer-signals as input, and provides the estimated heart rate as output. Finally, we compare the novel deep learning approach to classical methods, performing evaluation on four public datasets. We show that on large datasets the deep learning model significantly outperforms other methods: The mean absolute error could be reduced by 31% on the new dataset *PPG-DaLiA*, and by 21% on the dataset *WESAD*.

## 1. Introduction

For an increasing number of people, wearables are becoming essential in daily life. One application area is continuous heart rate monitoring, an important task in e.g., the healthcare or fitness domain. Electrocardiography (ECG) is a precise method of determining the heart rate, but is cumbersome in daily life settings. Therefore, wrist-worn heart rate monitors have become widely used recently. Heart rate monitoring is based on photoplethysmography (PPG) in these devices. Various commercial products, such as the Apple Watch [1], the Fitbit Charge [2], or the Samsung Simband [3], include a PPG-sensor nowadays. However, compared to the traditional ECG data, heart rate estimation is more challenging based on the PPG-signal. Especially motion artefacts, caused by the user’s physical activity, can significantly reduce the PPG-signal quality. Therefore, increasing the accuracy and robustness of PPG-based heart rate estimation remains at the forefront of research in this area.

One major issue within this research field is that existing public datasets are limited and they exhibit several major shortcomings. Since our goal is the mobile assessment of heart rate, particularly while affected by motion artefacts, PPG and accelerometer data is needed. State-of-the-art publications rely mostly on the two datasets introduced for the IEEE Signal Processing Cup in 2015 [4,5]. However, these datasets are rather short (contain less than one hour of data each), were recorded during only a few different physical activities, and data collection was performed under controlled conditions (lab-like setting). More recent datasets (such as the one introduced by Jarchi and Casson [6], or WESAD [7]) only partially address these shortcomings. Therefore, in order to develop and evaluate novel PPG-based heart rate estimation approaches (especially focusing on motion artefact compensation algorithms), an adequate benchmark with naturalistic data is required. We introduce a new publicly available dataset in this paper: *PPG-DaLiA*, which stands for *PPG dataset for motion compensation and heart rate estimation in Daily Life Activities*. This dataset includes in total more than 36 h of data, recorded from 15 subjects. The data collection protocol includes eight different types of activities (which are typically performed in daily life) and transition periods in between. Subjects performed these activities under natural, close to real-life conditions.

**Figure 1:**
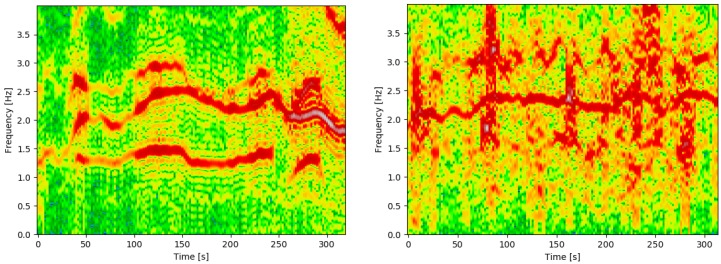
Time-frequency spectra of PPG-signals, derived from the IEEE datasets. Left plot: *IEEE_Training* dataset, session #8. Right plot: *IEEE_Test* dataset, session #6 (subject 5, exercise type 2).

Considering state-of-the-art PPG-based heart rate estimation approaches, Biswas et al. [8] provide a comprehensive overview. There exists a large amount of work relying on different (adaptive) filtering methods [9,10] or separating the heart rate component from the motion artefact and other noise components within the time-series [11,12]. However, recent work focuses on signal representations outside the time-domain, which proved to be beneficial to highlight the periodicity of the heart rate. For example, Jaafar and Rozali [13] applied wavelet transformation to estimate the heart rate and breathing rate from the PPG-signal. Another promising, widely-used signal representation is the time-frequency spectrum. Figure 1 shows example PPG-spectra, derived from the IEEE datasets. The periodic heart rate, as well as periodic physical activities are emphasised in these spectra. For example, considering the left plot in Figure 1, three dominant frequency components can be observed, from which the middle one represents the heart rate. The core idea of spectrum-based approaches is to distinguish the heart rate and motion-based periodicity, supported by motion-derived time-frequency spectra (e.g., based on simultaneously recorded acceleration signals). Based on such spectral analysis, several methods have recently been developed, such as IMAT [14], SpaMa [15], WFPV [16], and MC-SMD [17]. A few approaches are offline algorithms, incorporating post-processing of the heart rate estimates: SPECTRAP [18] or WFPPV+VD [19]. However, extracting the heart rate from time-frequency spectra is cumbersome with current approaches, as they are highly parametrised and were tailored to specific scenarios. The results presented in Section 3.3 indicate that there is still a need for an approach which generalises well.

The above listed approaches rely (at least partially) on hand-crafted rules and features. Instead, we suggest an end-to-end learning technique in this paper. Our deep learning approach takes the time-frequency spectra of the PPG- and accelerometer-signals as input, and provides the estimated heart rate as output. Deep learning has already been applied to various time-series signals, such as for human activity recognition [20,21,22] or gait parameter extraction [23]. Gjoreski et al. [24] compared deep and classical machine learning methods on the task of human activity classification, and showed that with sufficient data a convolutional neural network (CNN) outperforms classical models. Hammerla et al. [25] compared CNNs to recurrent neural networks (RNN), concluding that CNNs are more suitable for continuous periodic activities. As an example for a regression task, Hannink et al. [23] used CNNs to estimate different parameters related to human gait. Considering deep learning approaches for PPG-signal analysis, Shashikumar et al. [26] provide an example: They first applied wavelet transform on an 8-channel PPG-signal, and then used a CNN-model to extract relevant information, with the goal to detect atrial fibrillation. PPG-based ambulatory atrial fibrillation monitoring is the goal of Voisin et al. [27] as well: Taking directly the time-series signal as input, they use a 50-layer CNN (ResNeXt) to assess the presence or absence of atrial fibrillation. Furthermore, they used a tri-axial accelerometer (included in the wrist-worn wearable along with the PPG-sensor) to analyse the impact of motion artefacts on accuracy. Jindal et al. [28] perform PPG-based human identification through Deep Belief Networks and Restricted Boltzmann Machines. However, this approach requires distinctive features as input for the deep learning model, thus it is not an end-to-end learning technique. Biswas et al. [29] use the pre-processed time-series signal as input for a CNN-LSTM model to perform both PPG-based heart rate estimation and biometric identification.

In this paper, we use convolutional neural networks for PPG-based heart rate estimation, and compare our deep learning approach to state-of-the-art classical methods. Our approach provides an end-to-end learning technique, where the time-frequency spectrum of the PPG-signal and tri-axial acceleration serve as input. To the best of our knowledge, the newly introduced *PPG-DaLiA* is the largest, publicly available dataset for PPG-based heart rate estimation in close to real-life conditions. Consequently, the presented evaluation of both classical and deep learning approaches on this dataset is the most thorough in this field. Moreover, our novel deep learning approach significantly outperforms state-of-the-art methods, opening new directions within this research area. We would also like to point out that by evaluating methods on large datasets and performing leave-one-subject-out cross-validation, we obtain a good understanding of the generalisation capabilities of the different approaches. Overall, the main contributions of this work are the following:

1. We introduce and publish a new dataset called *PPG-DaLiA* for PPG-based heart rate estimation (The dataset introduced in this paper is made publicly available to support reproduction of and building on our research, and can be downloaded from: https://ubicomp.eti.uni-siegen.de/home/datasets/sensors19/). Our goal is to provide the research community a large dataset with a wide range of physical activities, performed under close to real-life conditions.
2. We introduce an extended version of the *SpaMa* algorithm, called *SpaMaPlus*. We show that on large-scale, realistic datasets it significantly outperforms two state-of-the-art classical approaches.
3. We introduce deep learning for PPG-spectrum-based heart rate estimation. We investigate different CNN-architectures and report insights based on our thorough evaluation.
4. We compare the novel deep learning approach to three classical methods, performing evaluation on four public datasets. We show that on large datasets our deep learning approach significantly outperforms classical methods which rely on hand-crafted rules and features. For example, the mean absolute error could be reduced by 31% on *PPG-DaLiA*.
5. Considering our deep learning approach, we discuss aspects of memory footprint and computational cost, and their relation in the trade-off with accuracy. Moreover, we propose a resource-constrained CNN-model consisting of only 26 K parameters, which still outperforms classical methods. These results demonstrate the practicability and feasibility of our CNN-based approach for heart rate estimation on wearable devices.

The above listed contributions have been partially presented in a workshop paper [30]. We briefly describe here the novelty of this manuscript with respect to previous work. The dataset *PPG-DaLiA* is a new, previously unpublished main contribution. *SpaMaPlus* was already introduced in [30], but algorithmic details were omitted there due to brevity. Our deep learning approach was introduced in [30] as well, but more details are provided in this manuscript. Moreover, evaluation was previously only performed on the two small IEEE datasets. We perform evaluation on two additional, larger datasets (*PPG-DaLiA* and *WESAD*) in this work, which provides a detailed and realistic estimate on the performance of each approach in real-life settings. Finally, the resource-constrained CNN-model is a new main contribution of this work, providing insights towards an embedded application.

## 2. Datasets

Although PPG-based heart rate estimation is widely used nowadays, the task of robust and continuous heart rate monitoring remains challenging. In order to develop, evaluate, and compare novel estimation approaches, publicly available datasets are essential. However, existing public datasets are limited and they exhibit several major shortcomings. We briefly review the available datasets in Section 2.1, and discuss their limitations. Moreover, in Section 2.2, we introduce a new publicly available dataset called *PPG-DaLiA*, short for *PPG dataset for motion compensation and heart rate estimation in Daily Life Activities*.

### 2.1. Available Datasets

Considering publicly available datasets, state-of-the-art PPG-based heart rate estimation approaches [5,15,30,31,32] mainly rely on the two datasets introduced as part of the IEEE Signal Processing Cup in 2015 [4,5]. This competition provided a training dataset (referred to as *IEEE_Training*) and a test dataset (referred to as *IEEE_Test*). Another recently published dataset focusing on heart rate estimation during physical exercises is the work done by Jarchi and Casson [6] (referred to as *PPG_Motion*). Furthermore, Lee et al. [33] provide another lab-recorded dataset for motion artefact cancellation in PPG-based heart rate estimation (referred to as *PPG_Bruce*). Finally, the dataset *WESAD* [7] includes data from various physiological sensor modalities, and can be used for PPG-based heart rate estimation as well. We briefly describe each of these four datasets in the subsequent paragraphs.

**IEEE_Training**: The dataset was recorded with a wrist-worn device, including a two-channel PPG sensor (green LED, wavelength: 515 nm) and a three-axis accelerometer (recorded at the same position as the PPG signal). Moreover, the dataset includes a simultaneously recorded ECG signal, providing heart rate ground truth. The sampling rate of all signals is 125 Hz. The dataset consists of 12 sessions, each recorded from a different subject (aged 18–35 years) while running on a treadmill at varying speed. Each session lasted approximately five minutes. In addition, the dataset provides mean heart rate on 8-second sliding windows (window shift: 2 s), extracted from the raw ECG signal. This derived signal can be used as ground truth. More details on this dataset can be found in [4,5].

**IEEE_Test**: The hardware setup as well as the provided raw and derived signals are the same as for the *IEEE_Training* dataset. However, the two datasets differ in the data collection protocol. The *IEEE_Test* dataset consists of 10 sessions, recorded from 8 different subjects (aged 19–58 years) while performing one of two exercise types. Subjects performed various forearm and upper arm exercises (such as arm rehabilitation exercises) during 4 sessions, and performed mainly intensive arm movements (such as boxing) during 6 sessions. Each session in the dataset lasted approximately five minutes. More details on this dataset can be found in [4,5].

**PPG_Motion**: This dataset was also recorded with a wrist-worn device including PPG, and an ECG sensor. However, instead of a single accelerometer, the wrist-worn device provides data from the following motion sensors: a 3-axis low-noise accelerometer, a 3-axis wide-range accelerometer, and a 3-axis gyroscope. Data was recorded from 8 subjects (aged 22–32 years), while performing the following physical activities: walking, running, easy biking, and hard biking. All of the activities were performed on exercise machines (treadmill and exercise bike), but the subjects could set the speed freely. Each activity lasted 3–10 min, but only one subject performed all four activities. Overall, subjects participated in the data recording with a mean duration of 14 min. More details on this dataset can be found in [6].

**PPG_Bruce**: Data from a wrist-worn device (including 3-channel PPG, 3-axis accelerometer, and 3-axis gyroscope) and simultaneously recorded ECG is provided in this dataset. Data was recorded from 24 subjects (10 male and 14 female, aged 26.9±4.8 years). Each subject followed a modified version of the Bruce protocol, consisting of the following activities (all performed on a treadmill): 2 min walking as warm-up, 3 min running, 2 min walking, 3 min running, and 2 min walking as cool-down. Thus, each session in the dataset lasted approximately 12 min. More details on this dataset can be found in [33].

**WESAD**: This is a multimodal dataset for wearable stress and affect detection. The dataset was recorded with a wrist-worn device (including the following sensors: PPG, accelerometer, electrodermal activity, and body temperature) and a chest-worn device (including the following sensors: ECG, accelerometer, EMG, respiration, and body temperature). 15 subjects (aged 24–35 years) participated in the data collection, each for approximately 100 min. The dataset was recorded with the goal to detect and distinguish different affective states (neutral, stress, amusement). Therefore, opposed to the above datasets, *WESAD* mostly includes data while subjects performed sedentary activities. More details on this dataset can be found in [7].

It should be noted that, for the above list of public datasets, we only considered datasets which include recordings from a wrist-worn PPG sensor (since wrist is the most important sensor location for heart rate monitoring in consumer devices, such as fitness bands or smart watches), a wrist-worn accelerometer (in order to facilitate motion artefact compensation), and simultaneously recorded ECG signal (in order to have access to ground truth data). Based on these criteria, the above list is comprehensive to the best of our knowledge, describing all relevant datasets for PPG-based heart rate estimation. Furthermore, since motion artefacts are considered the major challenge in this domain, the usefulness of *WESAD* is limited. Overall, we identify the following major shortcomings of existing datasets:

- Dataset size: while the number of subjects can be considered as sufficient (8–24 participants in each dataset), the length of each session’s recording is rather short. Therefore, the total duration of the datasets *IEEE_Training*, *IEEE_Test* and *PPG_Motion* is less than 120 min each. *PPG_Bruce* is the largest motion-focused dataset with nearly 5 h in total. *WESAD* is significantly longer (contains data of approximately 25 h). However, the challenging part of the dataset from the heart rate estimation perspective (thus, where motion artefacts are present) covers only a small fraction of it.
- Small number of activities: Each of the described public datasets includes data from only 2–3 different physical activities. Considering all datasets together, the following activities are included: walking, running, biking, boxing, arm exercises, and sedentary activities (sitting, standing). Different physical activities produce different motion artefacts within the PPG signal. Therefore, a larger variety of included activities would be beneficial to address a wider range of challenging signal artefacts.
- Data recording in laboratory settings: Each of the listed datasets was recorded in a lab-like setting, under controlled conditions. For example, locomotion activities (walking, running, biking) were performed on exercise machines. However, motion patterns in natural environments usually differ from motion performed in lab-like settings. Consequently, motion artefacts induced in the PPG signal differ as well. Moreover, transition-like activities are missing from the existing datasets, but take a significant part of daily life. Therefore, datasets recorded under natural conditions would be required to facilitate the deployment of developed heart rate estimation solutions into real-life settings.

### 2.2. New Dataset: PPG-DaLiA

Motivated by the limitations of existing public datasets, we introduce *PPG-DaLiA*: a *PPG dataset for motion compensation and heart rate estimation in Daily Life Activities*. Our goal is to provide the research community a large dataset with a wide range of physical activities, performed under natural, close to real-life conditions. Therefore, this new dataset can serve as a benchmarking dataset, which enables to evaluate and further explore the generalisation capabilities of PPG-based heart rate estimation approaches. In this section, we provide a detailed description of the new dataset, including hardware setup, participants, data collection protocol, ground truth generation, and various other characteristics of the dataset. The data collection user study was approved by the workers’ council and the data security officer of our research centre.

#### 2.2.1. Hardware Setup

Raw sensor data was recorded with two commercially available devices: a chest-worn device (RespiBAN Professional, [34]) and a wrist-worn device (Empatica E4, [35]). The RespiBAN Professional was used with the following sensor modalities: ECG, respiration, and three-axis accelerometer. All signals from the RespiBAN were sampled at 700 Hz. ECG signal was recorded via a standard three-point ECG, chosen as most suitable for our data collection protocol in the trade-off between restricting subject movement and providing high-quality ECG signal (one-point ECG has been proposed as an alternative measurement for long-term monitoring in case only the QRS complex is of interest [36]). Ag/AgCl electrodes were used and positioned as follows: The plus electrode was placed central on the right pectoralis major, the minus electrode on the lower left rib cage, and the ground electrode on the left pectoralis major. The RespiBAN device uses a 16-bit ADC, output is in the range of ±1.5 mV (with VCC=3 V). The output ECG signal is band-pass filtered: 0.5–100 Hz. The respiration signal was acquired with a respiration inductive plethysmograph sensor, embedded into the RespiBAN chest strap (16-bit ADC, device output is the displacement value in the range of ±50%). Three-axis acceleration was acquired via a 3D-accelerometer, integrated into the RespiBAN wearable device (16-bit ADC, signal range: ±3.6 g, output signal is low-pass filtered at 50 Hz). It should be noted that respiration and chest-worn acceleration signals are not directly relevant for PPG-based heart rate estimation. We only provide this data since these sensors are by default part of the RespiBAN device, and thus are recorded during any data collection. Nevertheless, we can imagine certain scenarios in the future where these sensor modalities become of interest as well (e.g., PPG-based respiration estimation, where data from the inductive plethysmograph can serve as ground truth).

The Empatica E4 device has been used in several user studies recently, with the goal to collect physiological data in real-life settings. For example, Gjoreski et al. [37] collected real-life data with the E4 to detect stress in unconstrained environments, or Di Lascio et al. [38] collected data during lectures to discriminate engaged from non-engaged students. Since the Empatica E4 proved to be reliable in long-term data recordings, we also used this device in our study to record PPG data. The E4 device was worn on the subjects’ non-dominant wrist. Data from the following sensor modalities is provided: PPG, three-axis acceleration, electrodermal activity, and body temperature. The PPG system consists of four LEDs (two green and two red) and two photodiode units (totalling 14 mm${}^{2}$ sensitive area). The PPG sensor output is the difference of light between oxygenated and non oxygenated peaks, provided with a sampling frequency of 64 Hz. Acceleration signal is in the range of ±2 g with 8-bit resolution, sampled with 32 Hz. Electrodermal activity signal is provided at 4 Hz, in the range of 0.01–100 μS and with 900 pS resolution. Body temperature signal is sampled at 4 Hz, with an accuracy of ±0.2 °C within 36–39 °C. Again, data of electrodermal activity and body temperature (both recorded by default) are not directly relevant for this work, but could be of interest for other usage of the dataset. Recorded data was stored locally on both devices during data collection, and transferred to a computer after the study protocol finished.

#### 2.2.2. Participants

All study participants were employees or students at our research centre. Exclusion criteria, stated in the study invitation, were cardiovascular diseases and pregnancy. In total, 15 subjects participated in the data collection: eight female and seven male participants, aged 21–55 years (mean and variance of age: 30.60±9.59 years). The dataset further provides information about each subject’s height, weight, skin type, and general fitness level. Skin type was classified according to the Fitzpatrick scale [39]. Fitness level is estimated based on the frequency of doing sports. All but two subjects stated to be in good to very good physical shape. Subject-level details can be found in the dataset itself, as well as in the documentation attached to it.

#### 2.2.3. Data Collection Protocol

With the wearable hardware setup described above, it is be possible to collect data directly from subjects’ daily life. However, this would result in long periods of data recordings with a rather small variety of different activities performed. Moreover, our hardware setup does not enable data collection during very intensive activities (such as running or rope jumping), since the strong motion artefacts induced in the recorded ECG signal would hinder the recovering of heart rate ground truth. Therefore, we decided to define a data collection protocol, which was followed by each subject. The protocol includes eight different activities, which are typically performed in daily life. We included activities of low (e.g., driving), medium (e.g., walking), and high-intensity arm movements (e.g., table soccer), in order to generate motion artefacts at diverse amplitudes. Another inclusion criterion was to have both periodic (e.g., walking or descending stairs) and aperiodic physical activities (e.g., eating or table soccer). Furthermore, in order to generate highly variable heart rates, we selected activities requiring different physical effort (e.g., driving vs. ascending stairs).

Table 1 gives an overview of the defined data collection protocol. Subjects were instructed to carry out the entire protocol as naturally as possible. Moreover, our specification of each of the activities was as vague as possible. This was especially the case for the two complex activities *lunch break* and *working*. For example, we only gave the instruction “*You have now 20 min for working*”, after which subjects returned to their workspace and continued working as if not participating in the study. The activities’ duration in the protocol is an approximate specification. Subjects were allowed to deviate from this in case a task was finished earlier or took longer. We briefly describe below each of the activities included in the protocol.

**Sitting**: Subjects were sitting still and reading (either on their laptop or provided magazines). The aim of this activity was to generate a motion-artefact-free baseline.

**Ascending and descending stairs**: Subjects were climbing six floors up and going down again, repeating this twice. This activity was carried out in the main building at our research campus.

**Table soccer**: Subjects were playing a one-on-one game with the supervisor of the data collection. While this is not an activity of regular daily life, we included table soccer due to the involved sudden and strong aperiodic motions, which we expect to produce challenging motion artefacts.

**Cycling**: Subjects performed this activity outdoors, around our research campus. They followed a defined route of about 2 km length with varying road conditions (gravel, paved). Subjects could freely choose their speed during this activity.

**Driving**: This activity started at the parking ground of our research campus and was carried out within the area nearby. Subjects were driving a common passenger car. Subjects followed a defined route which took about 15 min to complete. The route included driving on different streets in a small city as well as driving on country roads.

**Lunch break**: This activity was carried out at the canteen of our research campus. The activity included queuing and getting food, eating, and talking at the table.

**Walking**: This activity was carried out within the premises of our research campus. Subjects were walking back from the canteen to their workplace, with some detours in case the distance was too short.

**Working**: Subjects returned to their desk and worked as if not participating in this data collection protocol. For each subject, work mainly consisted of working on a computer.

**Figure 2:**
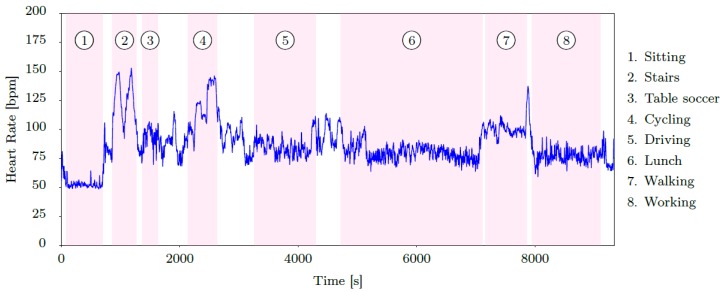
Data collection protocol: example recording of subject S7. *X*-axis: activities and transient periods performed during the protocol. *Y*-axis: heart rate, extracted from the ECG signal.

A transient period was included between most activities of the data collection protocol. These transitions were used to arrive at the starting location and prepare the next activity (e.g., walk to the parking ground and adjust the car settings before driving). Transient periods are labelled separately in the dataset. The data collection protocol took approximately 150 min for each subject. We have only encountered one major hardware issue, due to which the recorded data of one subject is only valid for the first 90 min. In total, *PPG-DaLiA* includes data of approximately 36 h, which can be used for PPG-based heart rate estimation. Figure 2 provides a concrete example of the data collection protocol, showing the sequence of activities as performed by subject S7. This figure also provides heart rate information extracted from the ECG signal (details given in the next subsection), which will serve as ground truth for PPG-based heart rate estimation.

#### 2.2.4. Data Synchronisation and Ground Truth Generation

The data collection protocol (labels of the activity and transient periods) was synchronised with the RespiBAN device during data recording. However, the Empatica E4 device required manual synchronisation to the RespiBAN. Therefore, during data collection, subjects performed a double-tap gesture with their non-dominant hand (where they wore the E4) on their chest. The double-tap gesture was performed both at the beginning and at the end of the data collection. The resulting characteristic acceleration signal patterns were used to manually synchronise the two devices’ raw data, performed in two steps. First, the double-tap at the beginning of the data collection was used to align the start time of the two devices. Second, the double-tap at the end of the data collection was used to correct time drift.

**Figure 3:**
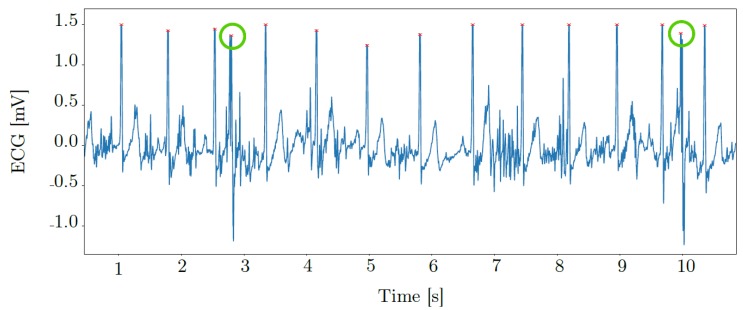
Example ECG-signal snippet from the data recording of subject S1. The two encircled R-peaks were falsely identified by the R-peak detector, and were thus manually removed during heart rate ground truth generation.

The main purpose of our new dataset *PPG-DaLiA* is to facilitate PPG-based heart rate estimation. Considering this task, reliable ground truth information can be obtained from the ECG signal. First, we used a well-known R-peak detector [40]. We then manually inspected and corrected identified R-peaks. This step was necessary in a few cases for each subject, due to severe motion artefacts within the ECG-signal. R-peak correction included both the removal of spurious peaks as well as recovering R-peaks missed in the first step. Figure 3 shows an example snippet of the ECG-signal of subject S1, where motion artefacts induced during the activity *table soccer* led to falsely identified R-peaks. Based on the identified and corrected R-peaks, we computed the instantaneous heart rate. Finally, the ECG-signal was segmented with a shifted window approach (window length: 8 s, window shift: 2 s). We defined ground truth heart rate as the mean instantaneous heart rate within each 8-s window. Applying this sliding window approach has been common practice in recent related work on PPG-based heart rate estimation [5,15,30,31,32].

#### 2.2.5. Dataset Characteristics

Overall, the dataset *PPG-DaLiA* includes the following synchronised signals: raw data from the RespiBAN (ECG, three-axis acceleration, respiration) and the Empatica E4 devices (PPG, three-axis acceleration, electrodermal activity, body temperature), ground truth heart rate, and activity information. The provided ground truth information (mean instantaneous heart rate on 8-second segments) enables easy comparison of various PPG-based heart rate estimation approaches. However, in case ground truth needs to be defined otherwise in a future usage of the dataset, the included raw sensor data from the chest-worn device enables this as well. Activity information is provided to better understand raw sensor data and induced motion artefacts.

In the remainder of this section, we provide further insights to *PPG-DaLiA*, and compare the new dataset to *IEEE_Training* and *IEEE_Test*. This comparison is motivated by the fact that the two IEEE-datasets are currently the most widely used for PPG-based heart rate estimation. Table 2 provides an overview about the heart rate distribution of each dataset. One sample refers to one 8-second segment of the data, for which a label (heart rate ground truth value) is defined in the dataset. A few observations can be made based on this table. First, due to the new dataset’s larger size (approximately 65 k samples vs. 1.8 k/1.3 k samples in the IEEE-datasets), *PPG-DaLiA* provides significantly more samples in each heart rate range than the two IEEE-datasets combined. Second, the new dataset offers a wider range of heart rate ground truth values (41–187 bpm) than the IEEE-datasets (68–176 bpm and 59–172 bpm for *IEEE_Training* and *IEEE_Test*, respectively). Third, the focus heart rate range differs between the three datasets. *IEEE_Training* mainly consists of walking and running activities, thus providing samples of intermediate to high physical effort. *IEEE_Test* has one focus on the lower intensity levels (rehabilitation arm movements) and a second focus on the higher intensity levels (boxing activities). Our new dataset *PPG-DaLiA* focuses on daily life activities, thus the main part of the dataset is labelled with low-medium heart rate values (60–120 bpm).

Table 3 provides session-wise details on heart rate statistics. The *IEEE_Training* dataset, as described above, consists of sessions with intermediate to high intensity level physical activities. Considering *IEEE_Test*, the two types of sessions are clearly visible in the heart rate statistics: Sessions S1, S2, S7, and S10 were recorded during arm rehabilitation exercises (rather low intensity activities), while the remaining sessions required much higher physical effort from the subjects. Considering our new dataset *PPG-DaLiA*, the heart rate statistics are similar across subjects. Subject S5 is a clear outlier, since mean heart rate is significantly higher than all other subjects. Considering subject S6, the main reason for the elevated mean heart rate compared to the majority of subjects is the fact that this is the subject for which part of the data is not available, and the missing part (approximately the last hour of the data collection protocol) consists of lower intensity activities than the available part. Overall, these insights into the datasets will explain the performance differences between sessions and datasets, presented in later sections of this work.

## 3. Classical Methods

We provide a brief overview of classical approaches for PPG-based heart rate estimation in this section. We implemented two state-of-the-art methods, and introduce a novel variant of an available algorithm in Section 3.1. We describe the evaluation metric used throughout this work in Section 3.2, and elaborate on evaluation schemes. Finally, in Section 3.3, we evaluate the three algorithms on four publicly available datasets (the two IEEE-datasets, *WESAD*, and *PPG-DaLiA*) and discuss results.

### 3.1. Algorithms

The datasets introduced for the IEEE Signal Processing Cup in 2015 triggered the development of various techniques in recent years, with the goal to estimate heart rate from PPG signals corrupted by motion artefacts. First, the authors of the IEEE-datasets introduced TROIKA [32] and JOSS [5]. Further methods followed within a short time, most relying on spectral analysis: WFPV [16], IMAT [14], MC-SMD [17], etc. A few methods are offline, thus post-processing heart rate estimates, for example, SPECTRAP [18] or WFPV+VD [19]. We disregard these methods in our analysis, since their practical application is limited, they do not enable direct feedback to the user in real-life scenarios. Instead, we focus on two recent algorithms in this work. First, the approach introduced by Salehizadeh et al. [15] (referred to as *SpaMa*-approach) was reported to outperform most online approaches (or at least perform similarly to other methods, see results in [15,19]). Second, the approach introduced by Schaeck et al. [31] (referred to as *Schaeck2017*-approach in this work) was reported to perform similarly while at lower computational cost than other methods. Moreover, we extend the heart rate tracking part of the *SpaMa*-algorithm, which will be referred to as the *SpaMaPlus*-approach. We will use these three algorithms (*SpaMa*, *SpaMaPlus*, and *Schaeck2017*) as classical methods for evaluation and comparison to deep learning approaches in this work. A brief description of these three classical algorithms follows below.

**SpaMa**: This approach first calculates the power spectral density of the PPG and accelerometer signals of each sample (8-second segment). Then, the highest peaks are found in each spectrum. The idea is that the peaks of the acceleration-spectrum correspond to motion. Therefore, removing these peaks from the PPG-spectrum results in removing the major motion artefacts. The highest peak in the remaining PPG-spectrum corresponds then to the heart rate. Algorithmic details of this approach can be found in [15].

**SpaMaPlus**: Since abrupt changes in heart rate are physiologically limited, relying on the estimated values from preceding segments is recommendable. However, the *SpaMa*-approach only considers the last segment in its heart rate tracking step. In order to increase the robustness of the estimation, we extended the heart rate tracking step with a mean filter over the last six heart rate estimates (the parameter six was empirically determined). We then consider the peak in the current segment’s PPG-spectrum closest to the heart rate frequency predicted by the mean filter. A second issue of the *SpaMa*-algorithm is that an error made in one segment’s heart rate estimation can propagate over a long time. *SpaMaPlus* overcomes this issue by resetting the heart rate tracking in case the difference between heart rate estimation of the current and previous segment exceeds a trainable threshold (e.g., 10 beats-per-minute) for an adjustable number of times (e.g., three times) consecutively. This way, heart rate estimation and tracking can recover every few seconds, even if spectral filtering completely fails over certain periods.

**Schaeck2017**: Opposed to *SpaMa* and *SpaMaPlus*, this approach supports the use of multiple PPG signal channels (the IEEE-datasets include two PPG channels). The algorithm first applies correlation functions (auto- and cross-correlation) on the time series signal. The aim of this step is to reduce noise. Then, the spectrum of the resulting time series is computed. Similar to the *SpaMa*-approaches, spectral filtering is applied to reduce motion artefacts by taking the acceleration spectra into account. Finally, a linear least squares fit on the preceding three segments is applied for heart rate tracking. Algorithmic details of this approach can be found in [31].

### 3.2. Evaluation Metric and Schemes

As described in Section 2, available datasets typically provide ground truth information for heart rate estimation. The instantaneous heart rate can be determined based on the recorded ECG signal. Then, ground truth is given every two seconds, defined as mean instantaneous heart rate over an 8-second segment of the data. Therefore, the goal of PPG-based heart rate estimation is to provide accurate heart rate values for the same segments. We adapt this sliding window approach (window length: 8 s, window shift: 2 s) for heart rate estimation, as was done in previous work [5,15,30,31,32]. Ground truth and heart rate estimation are given in beats-per-minute (bpm) in this work. As performance metric, we rely on the mean absolute error (MAE), defined as:

(1) $$MAE=\frac{1}{W}\sum_{w=1}^{W}BPM_{est}\left(w\right)-BPM_{ref}\left(w\right)$$

where *W* is the total number of windows, and $BPM_{est}\left(w\right)$ and $BPM_{ref}\left(w\right)$ denote the estimated and reference heart rate value in beats-per-minute on the *w*th window, respectively. This performance metric is commonly used in related work [5,15,31,32].

We implemented each of the three above described approaches: *SpaMa*, *SpaMaPlus*, and *Schaeck2017*. All three algorithms have in common that they include several adjustable parameters, such as the number of highest peaks to consider in the PPG and acceleration spectra, or the minimum required frequency difference for removing motion-induced peaks in the PPG spectrum. It is common practice in related work to tune these parameters for each dataset-session specifically, instead of applying a cross-validation scheme (e.g., see evaluation results for *SpaMa* [15] and *Schaeck2017* [31]). As a consequence, session-optimised results are reported with typically very low MAE-values. However, the practical relevance of these results is limited in our opinion. When deploying PPG-based heart rate estimation algorithms in real-life settings, there is no access to ground truth information (using an off-the-shelf chest-worn heart rate monitor would defeat the purpose of wrist-worn heart rate estimation, while only providing an approximate ground truth value with an unknown averaging method). Therefore, the optimisation of these algorithms to a specific subject or even a specific session is not possible in daily life. Instead of reporting session-optimised results, we argue that leave-one-session-out (LOSO) cross-validation should be preferred. During this cross-validation scheme, parameter optimisation is performed on all data except of one session, and the left-out session is used as test data. This procedure is repeated so that each session is used as test data exactly once. Thus, assuming a dataset with adequate (population, situation, etc.) variety, results reported with LOSO cross-validation can reflect the generalisation capabilities of the developed algorithms.

### 3.3. Evaluation Results

First, we evaluated each algorithm on the IEEE-datasets. For parameter setting, we performed both session optimisation and LOSO cross-validation, using random search of the parameter space in both cases. We report overall results in Table 4, session-wise results are given for *IEEE_Training* in Table 7 and for *IEEE_Test* in Table 8, respectively. Considering session optimisation, the results match the ones reported in the original publications. *SpaMa* achieved originally 0.89±0.6 bpm on *IEEE_Training* and 3.36±1.5 on *IEEE_Test* [15]; our results are: 1.33±1.4 bpm and 2.53±2.0 bpm, respectively. *Schaeck2017* achieved originally 1.32±1.2 bpm on *IEEE_Training* [31]; our result is 1.33±1.3 bpm. These small differences can be explained by the random parameter search. We observed that especially the *SpaMa*-based approaches are very sensitive to the parameter setting. As to be expected, considering LOSO cross-validation, the performance significantly decreased compared to the session-optimised results. Large performance difference between subject/session-dependent and -independent evaluation has been reported for example in the human activity recognition domain [41]. The optimised results with an MAE of only a few beats-per-minute could indicate that the task of PPG-based heart rate estimation is solved, even in scenarios with severe motion artefacts present. However, the session-independent results presented in this work show that the generalisation capabilities of existing algorithms are still limited. These findings again confirm the necessity of session-independent evaluation, otherwise presented results could be misleadingly optimistic.

**Figure 4:**
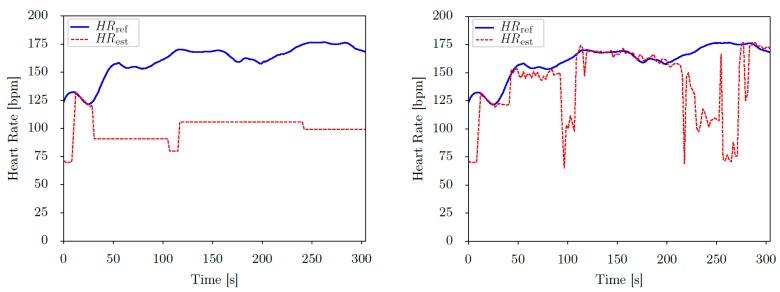
*IEEE_Training* dataset, session S10: comparison of ground truth ($HR_{ref}$) and estimated ($HR_{est}$) heart rate. Left plot: heart rate estimation with the *SpaMa*-approach, right plot: heart rate estimation with the *SpaMaPlus*-approach.

The three implemented algorithms performed differently on the two IEEE-datasets (see the results in Table 4). The *Schaeck2017*-approach performed best on *IEEE_Training*, but was significantly worse than the other two approaches on *IEEE_Test*. On the other hand, the best performing approach on *IEEE_Test* (*SpaMa*) was the worst on *IEEE_Training*. Overall, our modified version of the *SpaMa*-approach (*SpaMaPlus*) achieved the best combined result on both datasets while applying LOSO cross-validation (average MAE on the two datasets: 8.28 bpm). However, in case rapid changes in heart rate occur, *SpaMaPlus* performs typically somewhat worse than *SpaMa*, due to its averaging nature. This explains the difference between the two algorithms on *IEEE_Test*: 6 out of the 10 sessions of this dataset include intensive movements, thus rapid heart rate changes. On these sessions, *SpaMa* consequently outperformed *SpaMaPlus* (see Table 8 for session-wise results). A major improvement of *SpaMaPlus* over *SpaMa* is the faster recovery in case heart rate estimation and tracking is significantly inaccurate for a longer period of time. This behaviour is demonstrated in Figure 4, comparing the two algorithms’ estimation on session S10 of the *IEEE_Training dataset*. *SpaMa* forces heart rate tracking within defined boundaries, while *SpaMaPlus* enables to reset heart rate tracking, thus recovering heart rate estimation. This way, the MAE is reduced from 60.79 bpm to 21.28 bpm for this session (see Table 7). Nevertheless, session S10 remains the most challenging one within the *IEEE_Training* dataset.

Due to its practical relevance, as argued above, we will only focus on LOSO cross-validation in the rest of this work. Table 4 reports overall results on the datasets *WESAD* and *PPG-DaLiA*. Session-wise results for these two datasets are given in Tables 9 and 10, respectively. Several observations can be made based on these results. First, our *SpaMaPlus*-approach significantly outperforms the other two algorithms, on both datasets. Based on the arguments presented in Section 2, we consider *PPG-DaLiA* as the most relevant dataset when assessing algorithm performance for PPG-based heart rate estimation in daily life settings. *SpaMaPlus* achieves an MAE of 11.06 bpm on this dataset, which is an improvement of 29% compared to state-of-the-art results (*SpaMa* provides an MAE of 15.56 bpm). Second, the *Schaeck2017*-approach displays the poorest overall performance on these two datasets. This could be partially explained by the single-channel PPG data provided by both *WESAD* and *PPG-DaLiA*, thus preventing to use the noise-reducing cross-correlation component of the algorithm. On the other hand, *Schaeck2017* already exhibited a poor performance on the *IEEE_Test* dataset. Therefore, datasets with long or complex sessions seem to be problematic for this approach. A third observation is that, although *WESAD* is the least challenging dataset from the perspective of motion artefacts, heart rate estimation remains rather inaccurate (best result is an MAE of 9.45 bpm, achieved by *SpaMaPlus*). We explain this behaviour with the fact that all algorithms were developed with the focus on motion artefact compensation. In order to achieve better results on *WESAD*, other aspects might be more beneficial. Finally, our overall observation based on Table 4 is that there is still a need to develop novel heart rate estimation algorithms. As indicated by the LOSO cross-validation results, novel algorithms should exhibit better generalisation capabilities both considering different subjects as well as different situations (physical activities). It should be further noted that even session-optimised results are rather inaccurate on these two large datasets. We performed the same session-optimisation procedure on *WESAD* and *PPG-DaLiA* with the *SpaMaPlus*-approach as was done on the IEEE-datasets, achieving 7.48±2.1 bpm and 8.34±2.9 bpm, respectively. These results can be explained by the fact that both datasets consist of long sessions (100 and 150 min, respectively) with different activities performed. Thus, it is difficult to find a set of parameters which is optimal for an entire session.

## 4. Convolutional Neural Network Approach

We introduce and evaluate our deep learning approach in this section. Section 4.1 describes the model architecture (a convolutional neural network, CNN) in detail. We performed experiments to investigate the effect of various model hyperparameters, such as the number of layers or the size of the fully connected layer. These experiments were performed on the two IEEE-datasets, and are described in Section 4.2. We then used the best performing set of hyperparameters to train and evaluate a CNN-model on each of the two larger datasets as well (*WESAD* and *PPG-DaLiA*). Evaluation results and comparison to the classical methods are presented in Section 4.3.

**Figure 5:**
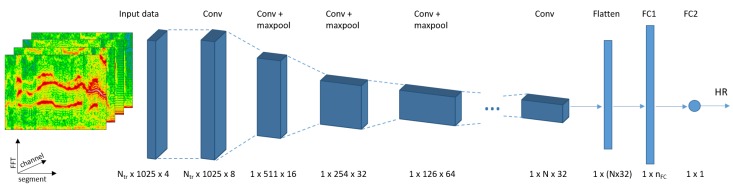
Proposed CNN-architecture with $N_{L}=1\cdots 8$ convolution-maxpool layers. $N_{tr}$ refers to the number of segments used together for heart rate tracking (see Section 4.1 for details). *N* depends on $N_{L}$. Input: $N_{tr}\times N_{ch}\times N_{FFT}$ matrix. Output: heart rate [bpm].

We perform a series of steps before data is fed to the deep learning model. First, we segment the time-series signal with the sliding window as defined before (window length: 8 s, window shift: 2 s, see Section 3.2 for details). We consider the first PPG-channel and all three accelerometer channels. Since some of the used datasets only provide single-channel PPG data, we focus on an approach which fits all datasets (similar to *SpaMa* and *SpaMaPlus*). However, as shown by the *Schaeck2017*-approach, relying on all available PPG-channels could improve the heart rate estimation accuracy. Therefore, investigating the benefits of multiple PPG-channels as input to our deep learning model remains for future work. As second step, we apply FFT on each time-series segment. The result of this step is $N_{ch}=4$ time-frequency spectra, one per signal channel. In the next step we cut these spectra, keeping only the 0–4 Hz interval (4 Hz corresponds to 240 bpm). The resulting number of FFT-points per segment and channel is $N_{FFT}=1025$. Finally, z-normalisation (zero mean and unit variance) is performed on each channel’s spectrum. The final $N_{ch}$ time-frequency spectra serves as input for the deep learning model (see Figure 5). Therefore, each 8-second segment of the original time-series is represented as a $N_{ch}\times N_{FFT}$ matrix. Furthermore, the ground truth of each segment is the heart rate value as provided by the datasets.

### 4.1. Model Architecture

We focus on convolutional neural network (CNN) architectures in this paper, as suggested in previous work on continuous periodic time-series analysis [21,23,25,27,42]. We investigated different network hyperparameters in a pre-study, such as the number of filters ($n_{f}$) and filter size ($size_{f}$) in each convolutional layer, activation function, stride in both the convolutional ($stride_{f}$) and the pooling layers ($stride_{p}$), size of the fully connected layer ($n_{fc}$), dropout-rate, loss function, optimisation approach, etc. Moreover, we incorporate tracking into the CNN model, since the estimated heart rate on a segment highly correlates to the preceding segments’ values, and heart rate tracking was beneficial in classical methods (*SpaMaPlus, Schaeck2017*). Therefore, the size of the input matrix for a segment changes to $N_{tr}\times N_{ch}\times N_{FFT}$, where $N_{tr}$ refers to the number of segments used together for heart rate estimation. The final architecture of our CNN model consists of the following layers (see Figure 5):

- convolution, $n_{f}^{1}=8$, $size_{f}^{1}=\left(1,1\right)$, $stride_{f}^{1}=\left(1,1\right)$
- convolution, $n_{f}^{2}=16$, $size_{f}^{2}=\left(N_{tr},3\right)$, $stride_{f}^{2}=\left(1,1\right)$
- max-pooling, $size_{p}^{2}=\left(1,2\right)$, $stride_{p}^{2}=\left(1,2\right)$
- for $i=1\ldots N_{L}$:
  - convolution, $n_{f}^{i}=2^{i+4}$, $size_{f}^{i}=\left(1,3\right)$, $stride_{f}^{i}=\left(1,1\right)$
  - max-pooling, $size_{p}^{i}=\left(1,2\right)$, $stride_{p}^{i}=\left(1,2\right)$
- convolution, $n_{f}^{last}=32$, $size_{f}^{last}=\left(1,1\right)$, $stride_{f}^{last}=\left(1,1\right)$
- flattening layer
- fully connected layer, with $n_{fc}^{1}$ neurons
- dropout layer, with dropout-rate: 0.5
- fully connected layer, with $n_{fc}^{2}=1$ neuron

The first convolutional layer performs fusion of the input channels (PPG and accelerometer). The second convolutional layer performs fusion of the segments involved in heart rate tracking. The subsequent convolution-pooling layers serve to increase the learning capabilities of the model, as demonstrated in Section 4.2. The last convolutional layer is included to reduce the input dimension to the fully connected layer, limiting model parameters and training time. Exponential linear unit (ELU) [43] is used as activation function throughout the entire model. The hyperparameters $N_{L}$, $n_{fc}^{1}$, and $N_{tr}$ will be further investigated in Section 4.2. Finally, the last fully connected layer outputs one value, the estimated heart rate. The loss function is defined as the absolute difference between this output and the respective segment’s ground truth value. Therefore, the goal is to optimise a regression problem. Optimisation is done using Adam [44].

Overall, our proposed model follows architectures from the image classification and object detection domains, where: (a) small 3×3 sized filters are preferably used, (b) the spatial size of the input volumes decreases after each layer (typically to the half), and (c) the depth of the volumes increases after each layer (the number of filters typically doubles). These principles have been successfully applied in well-known architectures, such as VGGNet [45], ResNet [46], or DenseNet [47]. One difference in our model is that the first spatial dimension shrinks to one after the second convolutional layer. Thus, we are using 1×3 sized filters in subsequent convolution steps. Our rationale of using an architecture similar to the ones used in image analysis is that a time-frequency spectrum can be regarded as a two-dimensional image. Therefore, defining features and extracting information of a spectrum could follow a similar process as applied in image analysis.

### 4.2. Experiments and Evaluation: IEEE-Datasets

We performed a thorough evaluation to investigate the effect of the following key hyperparameters of our CNN-architecture: $N_{L}$, $n_{fc}^{1}$, and $N_{tr}$. Moreover, we investigated the effect of applying ensemble prediction and batch normalisation. Our findings are reported below. All of these experiments were carried out on the IEEE-datasets. Due to their small size (1.8 k and 1.3 k samples, respectively), these datasets are suitable to experiment with a wide range of hyperparameters, as training time per experiment remains adequate. On the other hand, for the two larger datasets, convergence is usually only reached after several days of training. Therefore, for the datasets *WESAD* and *PPG-DaLiA*, we only apply the hyperparameter setting found to perform best on the IEEE-datasets.

As argued in Section 3.2, we will only focus on LOSO cross-validation while evaluating our deep learning approach. The applied evaluation scheme on the *IEEE_Training* dataset (consisting of 12 sessions) is as follows. The dataset is randomly split into 4 folds, each containing 3 sessions. 3 folds are used for training, while the remaining fold is split into validation (2 sessions) and test data (1 session). The validation-test split is rotated, then the same procedure is repeated on each of the 4 folds. Thus, training is performed in total 12 times, so that each session serves as test data exactly once. The applied evaluation scheme on the *IEEE_Test* dataset follows a similar procedure. Training is performed 10 times, with each of the 10 sessions serving as test data exactly once. The remaining part of the dataset is randomly split each time, having 7 sessions as training and 2 sessions as validation data.

All deep learning models described in this paper were implemented in TensorFlow version 1.3 [48]. Training and evaluation was done on Nvidia GTX 1080 TI GPUs with 12 GB of RAM and 1392 MHz of clock speed. For each test run, 15,000 training iterations with a batch size of 128 were performed, as this proved to be sufficient for reaching convergence on validation loss. For testing, we chose the model with the lowest validation loss. We repeated each experiment 7 times, randomly determining the training and validation sets each time.

**Figure 6:**
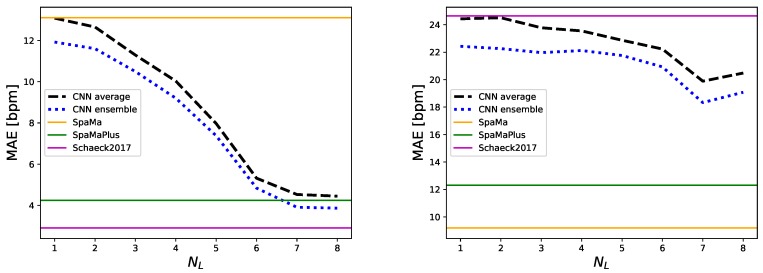
Evaluation results on *IEEE_Training* (**left**) and *IEEE_Test* (**right**) datasets, investigating the number of convolutional layers and ensemble prediction. LOSO results of the classical methods displayed for reference. $N_{L}$ refers to the hyperparameter defining the number of convolution-pooling layers, see Section 4.1.

**Number of convolutional layers**: We considered the range $N_{L}=1\cdots 8$ (corresponding to 3⋯10 convolutional layers in total when accounting for the first two layers of the architecture). Further convolutional layers (an even deeper model) were not considered, since both spatial dimensions shrink to one with $N_{L}=8$. For this experiment, $n_{fc}^{1}=512$ and $N_{tr}=7$ were fixed (best setting according to our pre-study). The evaluation results on both datasets are displayed in Figure 6. Overall, each additional layer increased the learning capabilities of the model, peaking at $N_{L}=7$. We do not have a satisfying explanation why the error increases on the *IEEE_Test* dataset when increasing the number of layers from $N_{L}=7$ to $N_{L}=8$.

**Ensemble prediction**: For each dataset session as test data, results from 7 repetitions (each providing a heart rate value per segment) are available. These were achieved with differently trained models, due to random initialisation and training-validation split. Therefore, since capturing more data variability during training, combining these models will lead to increased modelling capabilities [49]. We defined ensemble prediction as the mean value of the 7 repetitions. Results in Figure 6 show that combining the models decreases MAE by 6–14%. For example, with $N_{L}=7$: from 4.53 bpm to 3.91 bpm on *IEEE_Training* and from 19.89 bpm to 18.33 bpm on *IEEE_Test*, respectively.

**Size of the first fully connected layer**: We considered the range $n_{fc}^{1}=128\cdots 1024$, evaluation results on both datasets are shown in Table 5. This hyperparameter seems to have a marginal effect on the overall model performance. Thus, we kept $n_{fc}^{1}=512$ for the further experiments.

**Batch normalisation**: We added batch normalisation [50] after each convolutional and the first fully connected layer, results are shown in Table 5. While the effect on *IEEE_Training* is only marginal, a significant improvement can be observed on the *IEEE_Test* dataset: MAE was reduced from 18.33 bpm to 16.51 bpm (considering ensemble prediction). Thus, we included batch normalisation for the further experiments.

**Number of tracking segments**: We considered the range $N_{tr}=1\cdots 11$, results are shown in Table 6. This hyperparameter has also only a marginal effect on the model’s performance. Best performance was reached with $N_{tr}=3$ on the *IEEE_Training* dataset, and with $N_{tr}=7$ on the *IEEE_Test* dataset, respectively. However, one benefit of using a larger number of tracking segments is that heart rate estimation becomes smoother over time, which might be beneficial in practical applications. Thus, we consider $N_{tr}=7$ a good choice.

Overall, the best performing deep learning model is using the CNN-architecture as described in Section 4.1, incorporating batch normalisation and ensemble prediction, and applying the following hyperparameter setting: $N_{L}=7$, $n_{fc}^{1}=512$, and $N_{tr}=7$. Session-wise results, comparing the three classical methods to the best performing CNN model, are given in Table 7 and Table 8 for *IEEE_Training* and *IEEE_Test*, respectively. On the *IEEE_Training* dataset, the CNN-based result is comparable to the *SpaMaPlus*-approach (which provided the best combined results on the two IEEE-datasets, see Table 4). However, on the *IEEE_Test* dataset, *SpaMaPlus* outperforms the deep learning approach (MAE: 12.31 bpm and 16.51 bpm, respectively). We explain this with the fact that the *IEEE_Test* dataset includes recordings from different activities (such as boxing or arm rehabilitation exercises), while only consisting of 10 sessions. Therefore, especially for deep learning approaches, the available data per activity seems to be insufficient. We will show in the next section that on larger datasets our deep learning approach is not only comparable to the best performing classical methods, but significantly outperforms them.

### 4.3. Evaluation on Larger Datasets

We used the best performing hyperparameter setting, determined in Section 4.2, to train a CNN-model on each of the two larger datasets as well. This further evaluation of our deep learning approach is provided in this section, including a comparison of results to the classical methods. Session-wise results are given in Table 9 and Table 10 for the dataset *WESAD* and *PPG-DaLiA*, respectively. Several observations can be made based on these results. First, our CNN-based model significantly outperforms all classical methods: an improvement of 21% was achieved on *WESAD* (reducing MAE from 9.45 bpm to 7.47 bpm), and an improvement of 31% was achieved on *PPG-DaLiA* (reducing MAE from 11.06 bpm to 7.65 bpm). Second, ensemble prediction is clearly beneficial on these datasets as well: the mean absolute error could be reduced by over 10% on both datasets, compared to the average MAE of the 7 repetitions. Comparing the overall results on these two large datasets, a similar observation can be made as with the classical methods (see Section 3.3): the mean absolute error on *WESAD* (7.47 bpm) is only slightly lower than on *PPG-DaLiA* (7.65 bpm), although the dataset contains much less motion artefacts. We suspect that a different data representation than time-frequency spectra (which focuses on the periodicity of signal artefacts) would be beneficial to use as input for the deep learning model, but leave further investigations regarding this issue for future work.

**Figure 7:**
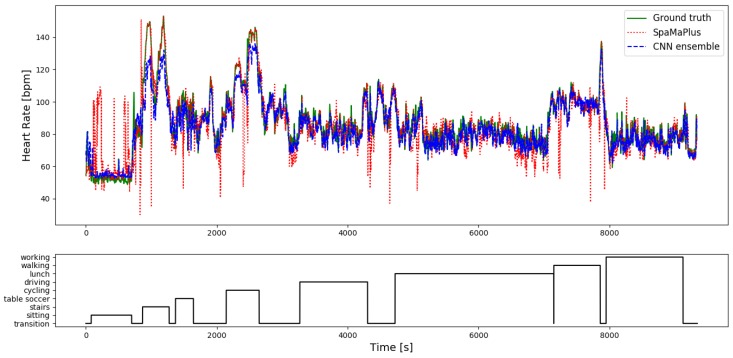
Heart rate estimation on the entire session S7 of the dataset *PPG-DaLiA*: Ground truth vs. prediction based on the *SpaMaPlus* approach vs. prediction based on our deep learning model (CNN ensemble). Activity labels are displayed in the lower subplot of the figure.

**Figure 8:**
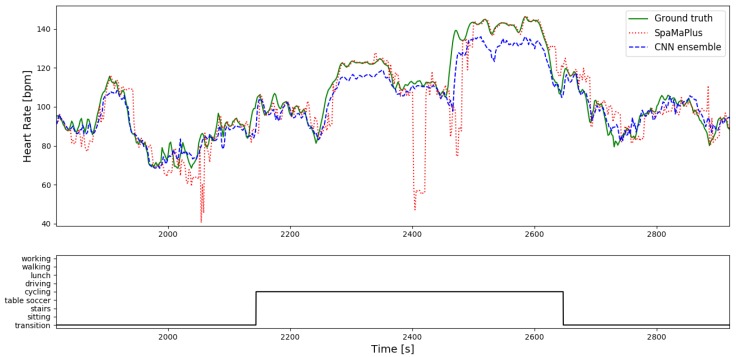
Heart rate estimation on the *cycling* part of session S7 of the dataset *PPG-DaLiA*: Ground truth vs. prediction based on the *SpaMaPlus* approach vs. prediction based on our deep learning model (CNN ensemble). Activity labels are displayed in the lower subplot of the figure.

Considering results on *PPG-DaLiA*, all above mentioned trends are followed (*SpaMaPlus* is the best performing classical method followed by *SpaMa* and then *Schaeck2017*; our deep learning approach outperforms the classical algorithms), but with an even larger margin. Figure 7 shows the result of heart rate estimation on the entire session S7 of the dataset *PPG-DaLiA*, comparing our CNN-approach and *SpaMaPlus* to the ground truth values. We would like to point out two observations based on this plot: (a) heart rate estimation with the deep learning approach leads to a smoother output, and (b) the deep learning based estimation follows the heart rate ground truth values rather well during each activity of the data collection protocol. Figure 8 zooms in on the *cycling* part of this session S7, in order to provide a more detailed view on the heart rate estimation. *SpaMaPlus* produces a few significant outliers within this part, while estimation with the deep learning approach remains within an acceptable range of error (below 15 bpm).

Results on the session S5 are by far the worst among all sessions on *PPG-DaLiA* (18.51 bpm with the CNN ensemble model, see Table 10). We explain this by the fact that subject S5 was a clear outlier in our data collection, since her mean heart rate was significantly higher than all other subjects. Therefore, the output of the training-validation process for session S5 results in an imprecise deep learning model. Interestingly, *Schaeck2017* performs better than any other approach on this session. The second highest MAE was achieved on the session S6 regarding our deep learning approach. However, this can be explained mainly by the fact that approximately the last hour of the protocol is not included in the dataset for this session, thus excluding large parts of the protocol (especially the activities *lunch* and *working*) which seem to be easier for heart rate estimation (see details below).

Finally, we provide insights on how well heart rate estimation performs on the different activities included in *PPG-DaLiA* (see Table 11). We would like to point out that the goal of this work is PPG-based heart rate estimation in general, regardless of the underlying physical activity. Therefore, when displaying results in this paper, each sample of the dataset is weighted equally. Nevertheless, in order to better understand the results and focus on improvement in future work, it is beneficial to know how well the different approaches performed on various activities. Based on Table 11, there is a strong correlation between the mean heart of an activity within the dataset and the achieved MAE on it. This observation was expected, since an elevated heart rate usually corresponds to increased physical activity, leading to larger motion artefacts (for example, the deep learning approach has an MAE of 16.98 bpm during the activity *stairs*). We also expected that *table soccer* will be a challenging activity for heart rate estimation, since it includes large aperiodic arm and hand movements (MAE of 12.16 bpm with the deep learning model). On the other hand, we expected a lower error during the activity *cycling*, since arm and hand movements are limited during this activity. Two possible explanations are that (a) nearly half of the route followed by the subjects during cycling was on gravel and (b) the wrist is somewhat bent during cycling (leading to artefacts caused by external light). However, further investigation is necessary to fully understand this part of the results. Considering the parts of the dataset consisting of mainly sedentary activities (*sitting*, *driving*, *lunch*, and *working*), both our deep learning approach as well as *SpaMaPlus* performed well.

### 4.4. Towards an Embedded Application

Having thus far presented a CNN-based approach that is agnostic to the system it is running on, we have significantly outperformed classical methods on large datasets. However, besides accuracy, the memory footprint (number of parameters) and the number of operations are crucial aspects for the practical realisation [51]. Our proposed CNN-model (hyperparameter setting: $N_{L}=7$, $n_{fc}^{1}=512$, $N_{tr}=7$) is very large and computationally expensive: a single model has 8.5 M parameters and requires 69.5 M computations for a single heart rate estimation. Considering an ensemble of seven CNN-models and inference every two seconds, the overall model requires approximately 60M parameters and 240M computations per second. On the other hand, PPG-sensors are most often used in wearable devices (such as smartwatches) with limited computational resources. Thus, a PPG-based heart rate estimation model would need to be deployed on embedded processors. Clearly, our proposed best performing CNN-model is computationally too expensive for such constrained devices. Therefore, we explore the trade-off between accuracy and computational cost in this section.

In order to find a suitable model in the trade-off accuracy and computational cost, we define the following goals. First, the model should follow a similar architecture to the one introduced in Section 4.1, since this CNN-architecture delivered high accuracy results in the previous sections. Second, we aim to outperform the classical methods even with a small CNN-model. More concretely, we aim to achieve a result better than MAE = 11.06 bpm on *PPG-DaLiA* (the most relevant dataset in our opinion). Third, we aim for a memory footprint of at most 32 KB. Certain embedded processors, such as the ARM Cortex series, are limited to hundreds or even less Kbytes. Moreover, these resources have to be shared among multiple processes. Thus, aiming to use maximum 32 KB for a heart rate monitoring application seems to be a realistic compromise, allowing to fit the CNN-model into a single RAM block. Recent advances in resource optimisation for deep learning models showed that using 8-bit quantisation is a good choice considering the trade-off accuracy and memory footprint. For example, Qiu et al. [52] showed that reducing bit width to 8-bit fixed point does not drop significantly the accuracy of GoogLeNet or VGG-16, compared to using 32-bit floating point or 16-bit fixed point. Therefore, assuming 8-bit quantised parameters and a maximum memory footprint of 32 KB, we aim for at most 32 K parameters in our CNN-model.

Reducing the number of parameters from 60 M to 32 K is substantial, thus requires to reduce and optimise the original CNN-model in many aspects. Compared to the CNN-architecture and best performing model described in the previous sections, we applied the following changes:

- $N_{FFT}=257$ (reduced from 1025), which reduces the input dimensions. Targeting a heart rate estimation resolution of 1 bpm seems sufficient in practical applications. Since we consider the frequency range 0–4 Hz, at least 240 FFT points are required. Considering the nearest power of 2 (for efficient FFT computation) and including both ends of the frequency range leads to 257 FFT points.
- $N_{tr}=1$ (reduced from 7), which reduces the input dimensions and makes the second convolutional layer obsolete (which performs fusion of segments for heart rate tracking). As shown in Section 4.2, providing several segments as model input had only a marginal effect on the accuracy. In case a smooth heart rate estimation would be beneficial for a practical application, this could be achieved by applying post-processing after the model’s output.
- $N_{L}=3$ (reduced from 7), thus reducing the number of convolution-pooling layers. A significant drop in accuracy is to be expected due to this change (see results presented in Section 4.2). However, reducing model size to 32 K parameters would not be possible while keeping $N_{L}=7$.
- $n_{f}^{last}=16$ (reduced from 32). This has the effect that the number of parameters in the first fully connected layer (which typically includes the most parameters in CNNs) is reduced by half.
- $n_{fc}^{1}=64$ (reduced from 512), thus reducing the number of parameters in the first fully connected layer further, by a factor of 8. As shown in Section 4.2, this hyperparameter had only a marginal effect on the overall model performance.
- Removed the dropout layer. This change has no effect on the memory footprint, but significantly increased accuracy during our explorative evaluations. We assume that the much smaller CNN-model does not include enough redundancy to justify a dropout layer.
- Only use a single CNN-model, instead of ensemble prediction. A significant drop in accuracy is to be expected due to this change as well (see results presented in Section 4.2). However, both the number of parameters and operations are directly reduced by a factor of 7 due to this change.

**Figure 9:**
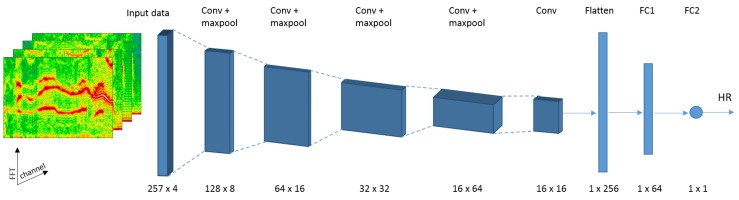
Proposed resource-constrained CNN-model. Input: 257×4 matrix (time-frequency spectra of the PPG-signal and three acceleration channels). Output: heart rate [bpm].

Therefore, the final resource-constrained CNN-model consists of the following layers (see Figure 9):

- convolution, $n_{f}^{1}=8$, $size_{f}^{1}=\left(1,1\right)$, $stride_{f}^{1}=\left(1,1\right)$
- max-pooling, $size_{p}^{2}=\left(1,2\right)$, $stride_{p}^{2}=\left(1,2\right)$
- for i=1…3:
  - convolution, $n_{f}^{i}=2^{i+3}$, $size_{f}^{i}=\left(1,3\right)$, $stride_{f}^{i}=\left(1,1\right)$
  - max-pooling, $size_{p}^{i}=\left(1,2\right)$, $stride_{p}^{i}=\left(1,2\right)$
- convolution, $n_{f}^{last}=16$, $size_{f}^{last}=\left(1,1\right)$, $stride_{f}^{last}=\left(1,1\right)$
- flattening layer
- fully connected layer, with $n_{fc}^{1}=64$ neurons
- fully connected layer, with $n_{fc}^{2}=1$ neuron

This resource-constrained CNN-model has 26 K parameters and requires 190 K computations per second. Therefore, it is a suitable model for embedded applications. The model achieves an MAE of 9.99±5.9 bpm and 8.2±3.6 bpm on the datasets *PPG-DaLiA* and *WESAD*, respectively (using LOSO cross-validation). Thus, it still significantly outperforms all classical methods on these datasets. The results of this resource-constrained model show that CNN-based heart rate estimation is not limited to large, computationally-expensive systems. Reducing the model size will impact in major advantages for implementation in embedded systems, with a moderate impact on its accuracy (see Table 12). This conclusion is in agreement with recent advances of deep learning becoming mainstream on constrained devices [53]. Moreover, further reduction of the CNN-model’s computational cost seems feasible while maintaining accuracy, considering recent work e.g., in pruning [54] or weight sharing [55]. Investigating such techniques for PPG-based embedded heart rate estimation models remains for future work.

## 5. Conclusions

We presented several contributions in this work to increase the robustness and generalisation capabilities of PPG-based heart rate estimation approaches. We introduced the new dataset *PPG-DaLiA* (*PPG dataset for motion compensation and heart rate estimation in Daily Life Activities*), which contains 36 h of data, recorded from 15 subjects (eight female and seven male participants, aged 21–55 years). The dataset will be publicly available for the research community, we encourage researchers to use it as a benchmark. Moreover, we argued that reporting session-optimised results has limited practical relevance, leave-one-session-out cross-validation should be applied instead. By using our newly introduced dataset and relying on a cross-validation evaluation scheme, a better understanding of the generalisation capabilities of different approaches can be obtained. However, *PPG-DaLiA* still has some limitations. The dataset does not represent all demographics, especially considering age, state of health, and skin type. Due to health care aspects, including elderly subjects would be of practical interest. Regarding state of health, collecting data from participants with cardiovascular issues would be important, since long-term heart rate monitoring is crucial for these subjects. With respect to skin type, participants in our data collection were in the range of II-IV on the Fitzpatrick scale (which ranges I-VI, where lower numbers refer to lighter skin [39]). Including participants with skin type V and VI would be important, since it is known that PPG-based heart rate monitoring is more challenging with dark pigmentation [56]. Finally, the number of subjects in *PPG-DaLiA* could be an additional limiting factor. Collecting data from significantly more subjects, while addressing the above demographic limitations, remains an important future task.

Considering algorithmic aspects, we showed that even with an improved version of a classical state-of-the-art method, the performance of heart rate estimation might not be satisfactory (the *SpaMaPlus* approach, which was the best performing classical algorithm throughout this paper, achieved a mean absolute error of only 11.06 bpm on the *PPG-DaLiA* dataset). Therefore, we introduced deep learning for PPG-based heart rate estimation in this work. Our end-to-end learning approach takes the time-frequency spectra of synchronised PPG- and accelerometer-signals as input, and provides the estimated heart rate as output. We investigated different convolutional neural network architectures and hyperparameter settings, and reported on insights (such as the benefits of batch normalisation and ensemble prediction) based on a thorough evaluation. On the two smaller IEEE datasets, our end-to-end learning approach did not perform better than classical methods. We explain this with the insufficient amount of available data per activity. However, when evaluating the deep learning approach on the two larger datasets, they significantly outperform all classical methods: an improvement of 21% was achieved on *WESAD* (reducing MAE from 9.45 bpm to 7.47 bpm), and an improvement of 31% was achieved on *PPG-DaLiA* (reducing MAE from 11.06 bpm to 7.65 bpm).

Overall, the topic of PPG-based heart rate estimation still needs further investigation, as indicated by the remaining inaccuracy during certain physical activities. Various input signal representations (time-domain, wavelet, or a combination of them with spectral representations) or deep learning architectures other than CNNs could be considered. Personalisation approaches, as defined in similar domains [57], could be beneficial as well. The accuracy and robustness of heart rate estimation could be further improved by exploiting the large amounts of unlabelled data (available since PPG-sensors can be easily worn during everyday life, while acquiring ground truth is more difficult). Considering practical aspects, we have to address the challenge of how to transfer the computationally expensive, deep CNNs to constrained devices (such as smart watches). We demonstrated a solution by proposing a resource-constrained CNN-model, showing that CNN-based heart rate estimation is feasible on wearable devices. Nevertheless, further investigation is required in the trade-off accuracy and computational cost, taking recent advances in resource optimisation for deep learning models into account.

Finally, it should be noted that this work is complementary to efforts done on the sensor technology level. For example, recent advances on the use of Silicon Photomultipliers (SiPM) are promising—an increased immunity of PPG systems to motion artefacts is expected [58].

## Figures and Tables

**Table 1:** Data collection protocol: activities and their duration.

Activity	Duration [min]
Sitting still	10
Ascending/Descending stairs	5
Table Soccer	5
Cycling	8
Driving car	15
Lunch break	30
Walking	10
Working	20

**Table 2:** Comparison of the datasets *PPG-DaLiA*, *IEEE_Training*, and *IEEE_Test*: Distribution of samples (8-s segments) with respect to heart rate ground truth.

Heart Rate [bpm]	PPG-DaLiA	IEEE_Training	IEEE_Test
0–40	0	0	0
40–60	3746	0	6
60–80	21,585	59	235
80–100	22,374	118	240
100–120	9884	248	153
120–140	4878	391	348
140–160	1679	696	247
160–180	512	256	99
180–200	39	0	0

**Table 3:** Comparison of the datasets *PPG-DaLiA*, *IEEE_Training*, and *IEEE_Test*: Session-wise details on heart rate statistics, in beats-per-minute. Note: considering the datasets *PPG-DaLiA* and *IEEE_Training*, sessions and subjects are interchangeable, since each subject performed exactly one session. Considering *IEEE_Test*, the 10 sessions were performed by only 8 subjects.

	PPG-DaLiA		IEEE-Training		IEEE-Test	
	Mean	Std	Mean	Std	Mean	Std
S1	74.84	16.16	133.41	30.18	74.73	10.00
S2	82.49	13.74	120.97	21.55	76.13	10.83
S3	89.01	15.85	131.28	21.21	127.69	17.82
S4	87.90	12.98	133.03	23.15	156.77	14.87
S5	125.84	19.56	141.93	19.09	122.81	12.57
S6	119.49	22.32	131.50	24.46	135.32	7.33
S7	86.69	18.55	134.61	20.19	90.61	14.13
S8	74.27	11.87	125.12	19.33	143.44	11.91
S9	87.26	19.95	126.07	22.62	127.34	8.53
S10	87.52	15.71	160.51	14.51	86.00	3.71
S11	102.67	23.52	152.49	18.10		
S12	71.41	14.81	141.66	21.88		
S13	89.21	22.05				
S14	89.64	18.69				
S15	79.66	17.84				
	89.43 ± 22.83		135.95 ± 24.30		115.39 ± 31.08	

**Table 4:** Evaluation of the classical methods on the datasets *IEEE_Training*, *IEEE_Test*, *WESAD*, and *PPG-DaLiA*. Session-optimised results reported only for the IEEE-datasets, results from LOSO cross-validation reported for each dataset. Results are given as MAE ± STD [bpm], where standard deviation is computed on the different individual sessions.

	IEEE_Training		IEEE_Test		WESAD	PPG-DaLiA
	Session-Optimised	LOSO	Session-Optimised	LOSO	LOSO	LOSO
SpaMa	1.33 ± 1.4	13.1 ± 20.7	2.53 ± 2	9.2 ± 11.4	11.51 ± 3.7	15.56 ± 7.5
SpaMaPlus	1.38 ± 1.4	4.25 ± 5.9	3.56 ± 3.9	12.31 ± 15.5	9.45 ± 2.9	11.06 ± 4.8
Schaeck2017	1.27 ± 1.3	2.91 ± 4.6	6.48 ± 8.3	24.65 ± 24	19.97 ± 8.1	20.45 ± 7.1

**Table 5:** Evaluation of size of the first fully connected layer ($n_{fc}^{1}$) and batch normalisation (BN). Average and ensemble prediction results are given, based on the 7 repetitions.

	IEEE_Training		IEEE_Test	
	CNN Average	CNN Ensemble	CNN Average	CNN Ensemble
$n_{fc}^{1}=128$	5.26	4.58	20.67	18.62
$n_{fc}^{1}=256$	5.04	4.41	20.79	19.64
$n_{fc}^{1}=512$	4.53	3.91	19.89	18.33
$n_{fc}^{1}=1024$	4.7	4.09	20.17	18.34
BN + $n_{fc}^{1}=512$	4.58	4	18.27	16.51

**Table 6:** Evaluation of the number of tracking segments ($N_{tr}$). Average and ensemble prediction results are given, based on the 7 repetitions.

	IEEE_Training		IEEE_Test	
	CNN Average	CNN Ensemble	CNN Average	CNN Ensemble
$N_{tr}=1$	4.86	4.3	18.99	17.39
$N_{tr}=3$	4.3	3.75	17.94	16.54
$N_{tr}=5$	4.44	3.89	18.39	16.86
$N_{tr}=7$	4.58	4	18.27	16.51
$N_{tr}=9$	4.27	3.76	20.56	18.52
$N_{tr}=11$	4.61	4.06	19.94	18.33

**Table 7:** Session-wise evaluation results on the dataset *IEEE_Training*, achieved with the three classical methods and the best performing CNN architecture (ensemble prediction). Results are given as MAE [bpm].

	S1	S2	S3	S4	S5	S6	S7	S8	S9	S10	S11	S12	All
SpaMa	51.41	19.21	1.66	1.9	5.05	3.13	3.79	2.05	1.93	60.79	2.33	3.92	13.1 ± 20.7
SpaMaPlus	3.19	9.97	1.61	2.74	1.5	2.82	1.03	2.22	0.41	21.28	1.84	2.44	4.25 ± 5.9
Schaeck2017	16	2.6	0.63	1.36	0.94	1.16	0.87	0.69	0.92	7.57	0.89	1.24	2.91 ± 4.6
CNN ensemble	8.83	3.56	2.37	3.04	1.66	2.05	1.04	1.63	1.33	19.72	1.62	1.09	4 ± 5.4

**Table 8:** Session-wise evaluation results on the dataset *IEEE_Test*, achieved with the three classical methods and the best performing CNN architecture (ensemble prediction). Results are given as MAE [bpm].

	S1	S2	S3	S4	S5	S6	S7	S8	S9	S10	All
SpaMa	18.26	2.35	9.77	37.52	5.43	2.3	2.58	10.81	2.5	0.44	9.2 ± 11.4
SpaMaPlus	17.54	3.37	13.7	53.33	9.05	2.08	2.72	13.06	7.39	0.87	12.31 ± 15.5
Schaeck2017	38.44	50.27	13.76	77.58	9.29	2.52	14.07	25.49	9.32	5.77	24.65 ± 24
CNN ensemble	37.29	12.17	9.26	52.54	10.09	3.67	14.12	18.4	3.61	3.91	16.51 ± 16.1

**Table 9:** Session-wise evaluation results on the dataset *WESAD*, achieved with the three classical methods and the best performing CNN architecture (ensemble prediction; results averaging the 7 repetitions are given for comparison). Results are given as MAE [bpm].

	S2	S3	S4	S5	S6	S7	S8	S9	S10	S11	S13	S14	S15	S16	S17	All
SpaMa	9.05	17.96	6.06	10.2	7.55	10.58	9.85	8.46	11.36	16.12	14.44	10.58	9.03	17.81	13.64	11.51 ± 3.7
SpaMaPlus	7.31	12.57	5.92	7.35	6.36	9.23	8.91	6.32	8.18	16.25	12.96	10.15	8.34	10.67	11.16	9.45 ± 2.9
Schaeck2017	23.3	37.09	26.15	30.54	18.57	21.29	19.55	13.26	20.74	16.72	9.78	6.32	16.55	12.6	27.09	19.97 ± 8.1
CNN average	5.94	14.75	8.84	8.7	4.58	7.44	5.56	5.61	9.44	11.68	7.92	6.75	5.58	13.51	10.03	8.42 ± 3
CNN ensemble	5.07	14.48	7.84	7.7	3.88	6.78	4.27	3.99	8.89	11.07	6.52	5.26	4.18	12.78	9.36	7.47 ± 3.3

**Table 10:** Session-wise evaluation results on the dataset *PPG-DaLiA*, achieved with the three classical methods and the best performing CNN architecture (ensemble prediction; results averaging the 7 repetitions are given for comparison). Results are given as MAE [bpm].

	S1	S2	S3	S4	S5	S6	S7	S8	S9	S10	S11	S12	S13	S14	S15	All
SpaMa	11.86	14.75	9.53	17.2	39.28	16.78	15.88	15.2	17.19	9.08	21.63	12.63	9.5	10.73	12.23	15.56 ± 7.5
SpaMaPlus	8.86	9.67	6.4	14.1	24.06	11.34	6.31	11.25	16.04	6.17	15.15	12.03	8.5	7.76	8.29	11.06 ± 4.8
Schaeck2017	33.05	27.81	18.49	28.82	12.64	8.72	20.65	21.75	22.25	12.6	21.05	22.74	27.71	12.05	16.4	20.45 ± 7.1
CNN average	8.45	7.92	5.96	7.86	18.97	13.55	5.16	11.49	10.65	6.07	9.87	9.95	5.25	5.85	5.25	8.82 ± 3.8
CNN ensemble	7.73	6.74	4.03	5.9	18.51	12.88	3.91	10.87	8.79	4.03	9.22	9.35	4.29	4.37	4.17	7.65 ± 4.2

**Table 11:** *PPG-DaLiA*: analysis of the dataset (number of samples and mean heart rate) and achieved results (with the best performing deep learning and classical approach) for each activity included in the data collection protocol.

	Number Samples	Mean HR [bpm]	MAE CNN Ensemble [bpm]	MAE SpaMaPlus [bpm]
Transition	17,495	94.72	8.77	14.34
Sitting	4573	61.07	4.93	4.27
Stairs	3242	118.53	16.98	25.42
Table soccer	2312	90.4	12.16	21.48
Cycling	3479	123.05	12.48	11.97
Driving	6845	84.7	4.96	6.24
Lunch	13,552	83.58	5.22	7.33
Walking	4697	99.18	9.21	18.16
Working	8502	76.4	3.84	4.91

**Table 12:** Comparison of the large, system-agnostic CNN-models (*CNN average* and *CNN ensemble*) and the resource-constrained embedded solution (*CNN constrained*). Performance results are given on the two large datasets (*PPG-DaLiA* and *WESAD*) as MAE [bpm]. Performance of *SpaMaPlus* shown as reference for best performing classical approach.

	Performance (MAE)		Computational Cost	
	PPG-DaLiA	WESAD	Number of Parameters	Operations per Second
CNN average	8.82 ± 3.8	8.42 ± 3	8.5 M	34.5 M
CNN ensemble	7.65 ± 4.2	7.47 ± 3.3	60M	240M
CNN constrained	9.99 ± 5.9	8.2 ± 3.6	26K	190K
SpaMaPlus	11.06 ± 4.8	9.45 ± 2.9		
